# Identification of NTCP animal orthologs supporting hepatitis B virus binding and infection

**DOI:** 10.1128/jvi.01833-24

**Published:** 2025-03-05

**Authors:** Fuwang Chen, Jochen M. Wettengel, Florian Gegenfurtner, Judith Moosmüller, Till Bunse, Samuel D. Jeske, Philipp Hagen, Yi Ni, Stephan Urban, Ulrike Protzer

**Affiliations:** 1 Institute of Virology, School of Medicine and Health, Technical University of Munich/Helmholtz Munich, Munich, Germany; 2 German Center for Infection Research (DZIF), partner site Munich 655317 , Munich, Germany; 3 Department of Infectious Diseases, Molecular Virology, University Hospital Heidelberg 27178 , Heidelberg, Germany; 4 German Center for Infection Research (DZIF), partner site Heidelberg 574554 , Heidelberg, Germany; University of Southern California Keck School of Medicine, Los Angeles, California, USA

**Keywords:** NTCP, HBV infection, HBV animal models

## Abstract

Hepatitis B virus (HBV) infection is a significant global health threat, resulting in more than 800,000 deaths annually. Since HBV naturally infects only humans and chimpanzees, the development and evaluation of new therapies for chronic HBV infection are hindered by the lack of suitable animal models. Human sodium-taurocholate cotransporting polypeptide (NTCP) is a critical factor for HBV binding and entry, exhibiting species-specific differences in the amino acid sequences. This study investigated NTCP orthologs from various species to determine their capability to support HBV binding and infection. We demonstrate that nonhuman NTCP orthologs from woodchuck, ferret, aardvark, horse, rabbit, whale, big brown bat, cat, and rhinoceros support HBV binding and cellular entry, thereby rendering HepG2 cells susceptible to HBV infection upon expression. NTCP orthologs from hamster, goat, and cow support HBV binding but require specific amino acid exchanges to facilitate HBV infection. We show that replacement of the functional region, amino acids (aa) 84–87, in hamster NTCP with the human counterpart allows infection of HepG2 cells expressing the chimeric NTCP variant. Furthermore, we demonstrate that aa 82 in goat and cow NTCP, close to this functional region, needs to be modified to support HBV infection. This study could help identify previously unknown HBV reservoirs and may facilitate the establishment of new animal models.

**IMPORTANCE:**

The *bona fide* HBV entry receptor NTCP provides a natural barrier for cross-species transmission. We identified species-specific NTCP orthologues from woodchuck, ferret, aardvark, horse, rabbit, whale, big brown bat, cat, and rhinoceros that support HBV infection. This may reveal potential HBV reservoirs and facilitate the development of new HBV animal models.

## INTRODUCTION

Worldwide, approximately 296 million individuals are affected by chronic hepatitis B (CHB) and face an increased risk of developing liver cirrhosis and hepatocellular carcinoma (HCC). In 2022, an estimated 1.1 million deaths were attributed to HBV-related diseases (1). Despite several achievements in the development of new antiviral therapies, reliable curative therapies for CHB remain unavailable.

HBV infection of hepatocytes is initiated by the attachment of the virus to glycosaminoglycan side chains of heparan sulfate proteoglycans (HSPGs) and the subsequent binding of the PreS1 domain of the large surface protein (L-protein) to sodium taurocholate cotransporting polypeptide (NTCP) (2–4). Human NTCP (hNTCP), encoded by the solute carrier family 10-member 1 gene (SLC10A1), is a transporter for hepatic bile acid uptake and has been identified as the *bona fide* HBV entry receptor essential for cellular binding and uptake (3–5). The discovery of hNTCP as the HBV entry receptor has been a landmark in HBV research as overexpression of hNTCP has enabled the establishment of HBV *in vitro* infection models, such as the HepG2-NTCP and Huh7-NTCP cell lines (3, 4, 6–8). These new cell culture models have facilitated the investigation of the HBV entry mechanism (8) and enabled high-throughput screenings for antiviral substances (9, 10).

Moreover, NTCP has been identified as a determining factor for HBV species specificity (11). In hNTCP, amino acid (aa) residues 157–165 have been identified as a critical motif for the PreS1–NTCP interaction (3, 4), and differences in these residues in macaque NTCP, for example, result in a complete block of HBV binding and infection (3, 12). Accordingly, cynomolgus and rhesus macaque hepatocytes expressing hNTCP or humanized macaque NTCP (aa 157–165) allowed for establishing HBV infection *in vitro* and *in vivo* (13, 14). While mouse NTCP sufficiently binds HBV, aa 84–87 are crucial for a post-binding step (15), presumably fusion or entry. However, the expression of hNTCP or a humanized murine NTCP variant in mice does not render mouse hepatocytes permissive (16, 17), suggesting other intracellular factors limit HBV infection (18). Therefore, identifying species-specific NTCP orthologs that support HBV binding and entry could help identify unknown HBV reservoirs and, if the species-specific hepatocytes support all post-entry steps of HBV infection, establish new HBV animal models.

Here, we selected 12 NTCP orthologs based on their similarity to the hNTCP-binding domain (aa 157–165). We analyzed their capability to render HepG2 cells susceptible to HBV and found that NTCP orthologs from aardvarks, rabbits, whales, big brown bats, cats, and rhinoceroses support HBV binding and infection upon expression. Moreover, hamster, cow, and goat NTCP can bind HBV but require additional aa changes to allow HBV infection.

## RESULTS

### Selection of NTCP variants based on sequence similarity with hNTCP aa 157–165

**Fig 1:**
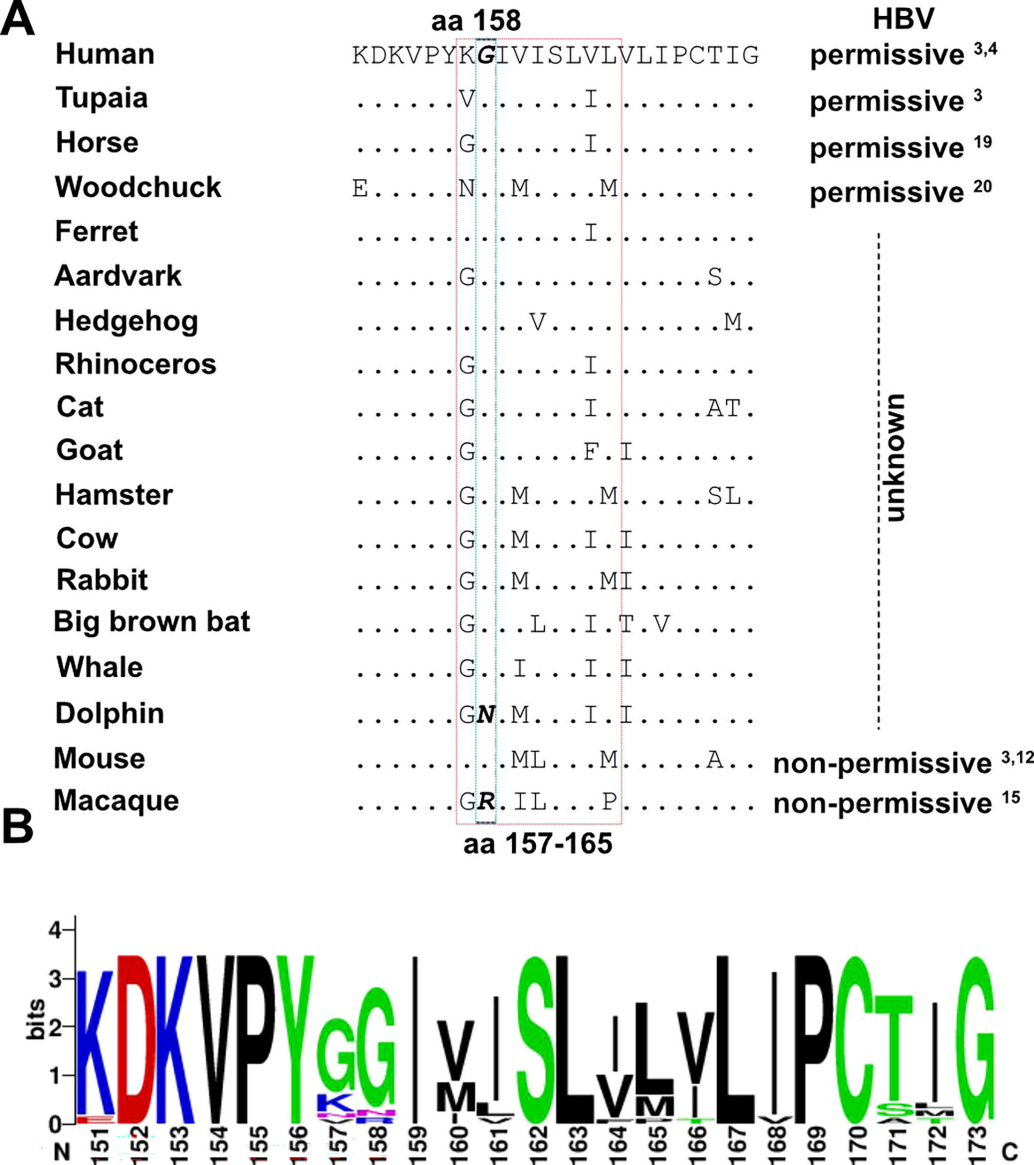
Selection of NTCP orthologs based on sequence similarity with hNTCP aa157–165. (**A**) Alignment of different NTCP orthologs at the binding domain (aa 157–165). Differences among aa 151–173 compared to hNTCP were indicated. HBV permissiveness, according to previous reports, is indicated. (**B**) Sequence conservation analysis among the NTCP orthologs concerning the HBV-binding domain.

To identify orthologs of hNTCP that allow HBV binding and infection, we retrieved species-specific NTCP sequences from public databases and selected them based on their amino acid sequence similarity to hNTCP aa 157–165 (Fig. 1A; Table 1). NTCP variants known to enable HBV infection upon expression from tupaias (3), horses (19), and woodchucks (20) served as positive controls. Macaque NTCP (3, 12), which does not mediate HBV binding, and mouse NTCP (15), which supports binding but not HBV cell entry, served as negative controls. Notably, among the selected sequences, residue G158 is conserved in all NTCP orthologs, except dolphin and macaque NTCP, while residues at aa positions 157, 160, 161, 164, and 165 in the HBV-binding motif are diverse (Fig. 1B).

**TABLE 1:** NTCP ortholog sequences tested in this study

ID no.	Accession no.	Size	Common name^*a*^	Scientific name	Order
1	NP_003040.1	349 aa	Human	*Homo sapiens*	Primates
2	XP_001110268.1	349 aa	Macaque	*Macaca mulatta*	
3	NP_001171032.1	362 aa	Mouse	*Mus_musculus*	Rodentia
4	XP_005072871.1	362 aa	Hamster	*Mesocricetus auratus*	
5	XP_015346065.1	348 aa	Woodchuck	*Marmota marmota*	
6	XP_005686078.2	370 aa	Goat	*Capra hircus*	Artiodactyla
7	NP_001039804.1	370 aa	Cow	*Bos taurus*	
8	XP_026967429.1	349 aa	Dolphin	*Lagenorhynchus obliquidens*	
9	XP_007184433.1	349 aa	Whale	*Balaenoptera acutorostrata scammoni*	
10	XP_003987831.1	349 aa	Cat	*Felis catus*	Carnivora
11	XP_004739077.1	372 aa	Ferret	*Mustela putorius furo*	
12	XP_001500613.1	349 aa	Horse	*Equus caballus*	Perissodactyla
13	XP_004426350.1	349 aa	Rhinoceros	*Ceratotherium simum simum*	
14	XP_008137122.1	348 aa	Big brown bat	*Eptesicus fuscus*	Chiroptera
15	NP_001076237.1	348 aa	Rabbit	*Oryctolagus cuniculus*	Lagomorpha
16	XP_007940520.1	349 aa	Aardvark	*Orycteropus afer afer*	Tubulidentata
17	XP_007535314.1	351 aa	Hedgehog	*Erinaceus europaeus*	Eulipotyphla
18	XP_006171565.1	379 aa	Tupaia	*Tupaia chinensis*	Scandentia

^*a*^ The common name of each mammal is shown in this study.

### Generation of an expression cassette for an indirect quantification of NTCP expression

**Fig 2:**
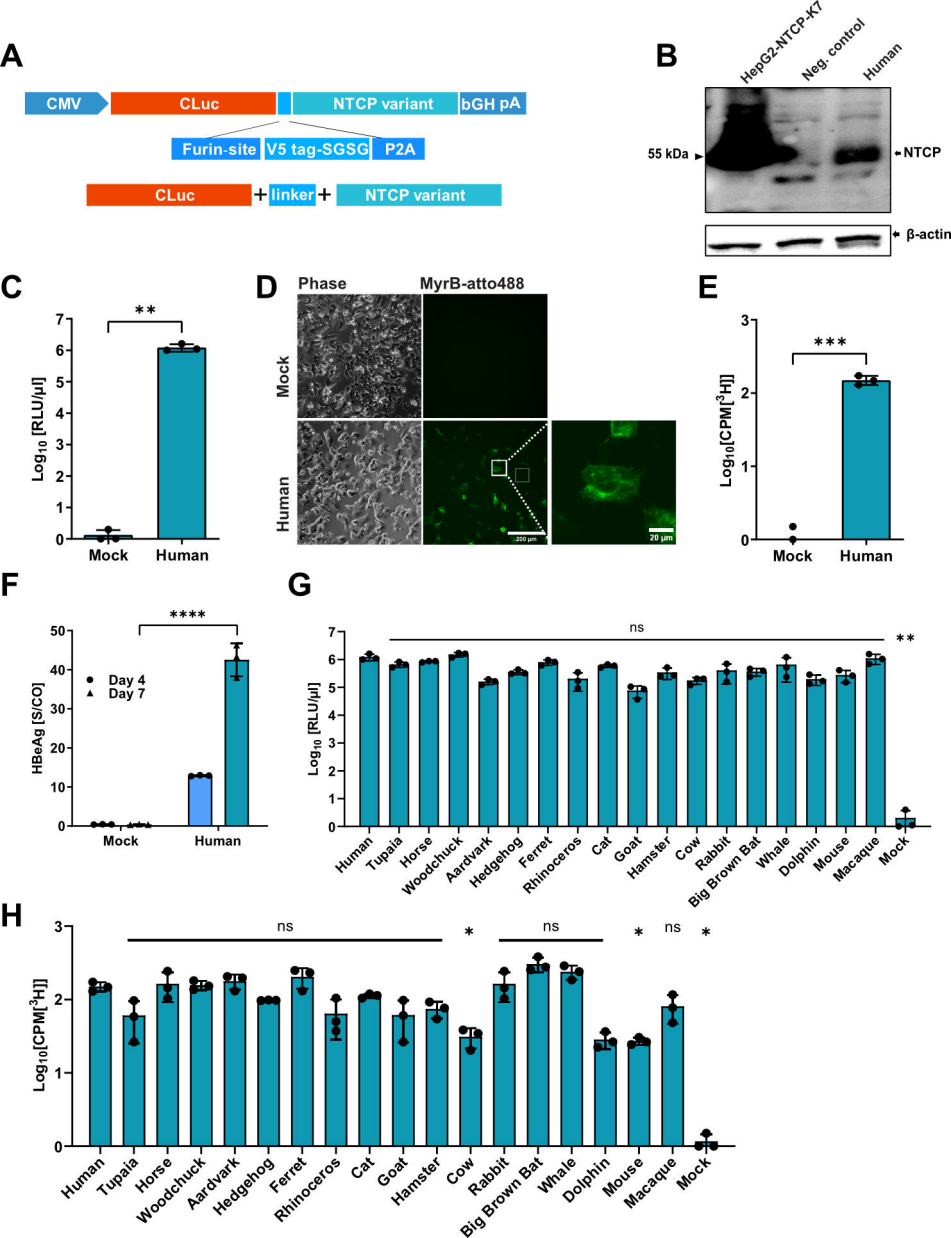
Generation and validation of an expression cassette for an indirect quantification of NTCP expression. (**A**) Schematic illustration of the expression cassette for an indirect quantification of the NTCP variant expression. Cluc and the respective NTCP variant are connected via a linker sequence containing a Furin site, a V5 epitope, an SGSG linker-sequence, and a T2A site. (**B–D**) HepG2 cells were transfected with the expression vector encoding hNTCP as the NTCP variant. Supernatants were analyzed at 3 days post transfection (dpt). Nontransfected cells served as a negative control. (**B**) Total hNTCP protein expression was detected by Western blot. HepG2-NTCP-K7 cells served as a positive control. (**C**) Determination of secreted Cluc in the supernatant of the transfected cells. (**D**) Transfected HepG2 cells were incubated with MyrB_atto488_ and analyzed via fluorescence microscopy for MyrB_atto488_ binding. (**E–F**) Transfected HepG2 cells were differentiated with 2.5% dimethyl sulfoxide (DMSO) for 2 days and inoculated with [^3^H]-taurocholate or HBV. (**E**) Uptake of [^3^H]-taurocholate was determined by a scintillation analyzer. HepG2 cells transfected with an expression vector without an NTCP variant served as mock. (**F**) Secreted HBeAg in the supernatant was measured at 4 and 7 dpi. Data represent one experiment as technical triplicates. (**G**) Corresponding NTCP ortholog expression was quantified indirectly by the measurement of secreted Cluc in the supernatant. (**H**) Transporter abilities of the NTCP orthologs were detected by the measurement of intracellular [^3^H]-taurocholate. Transporter abilities were shown by subtracting the background radioactivity values. Experiments were performed in biological triplicates; mean values +/– standard deviation are given. (**C, E**) Data were analyzed by unpaired *t*-test. (**F**) Data for 7 dpi were analyzed by unpaired *t*-test. (**G, H**) Data were compared to human control by one-way ANOVA with Dunnett’s multiple comparison correction. Statistical significance is denoted as follows: **, *P* < 0.01; ***, *P* < 0.001; ****, *P* < 0.0001; ns, not significant.

The selected NTCP sequences were codon-optimized, synthesized, and cloned into liver-specific expression vectors (14). Since there is limited availability of antibodies capable of directly detecting the expression of the species-specific NTCP orthologs, we decided to co-express Cypridina luciferase (Cluc) to allow an indirect quantification of the expression. As shown in Fig. 2A, the different NTCP sequences were linked to Cluc via a furin-V5-SGSG-T2A linker (21, 22), leading to an equimolar expression of the N- and C-terminal protein. Since the T2A linker peptide functions co-translationally, it leads to a minimally modified C-terminus of the Cluc and N-terminus of the respective NTCP variant. To verify the correct co-expression and complete cleavage of the two proteins, we transfected HepG2 cells with an expression plasmid encoding hNTCP and performed Western blot analysis on the cell lysates. Our results show that hNTCP is expressed at the same size as in the previously described stable cell line HepG2-NTCP-K7 (8) (Fig. 2B), indicating correct cleavage of Cluc and hNTCP. Next, we analyzed the functionality of both Cluc and hNTCP. Therefore, we first analyzed the supernatant of the transfected cells for luciferase activity and detected high signals compared to mock (Fig. 2C). We then evaluated the correct surface localization of the co-expressed hNTCP. For this experiment, we used an Atto488-fluorescently labeled myristoylated HBV-PreS1 peptide (MyrB_atto488_), which specifically binds to hNTCP (3, 4, 15, 23, 24). As expected, HepG2 cells co-expressing hNTCP bound MyrB_atto488_ on their membrane (Fig. 2D), indicating correct protein folding and surface localization. We further verified whether co-expressed hNTCP maintained its physiological function of bile acid uptake by analyzing intracellular [^3^H]-taurocholate uptake and detected increased levels of [^3^H]-taurocholate after 15-min incubation (Fig. 2E). Finally, we analyzed the function of the co-expressed hNTCP to support HBV infection. We detected secreted hepatitis B e antigen (HBeAg) at 4- and 7 days post-infection (dpi) (Fig. 2F), indicating that hNTCP is fully functional in our co-expression system.

In summary, these data show that our system leads to the correct expression of both proteins, the Cluc and the NTCP variant, allowing the indirect quantification of the NTCP variant expression.

### Analysis of species-specific NTCP orthologs for bile acid uptake and HBV binding and infection

To validate whether the NTCP orthologs maintained their functional properties as bile acid transporters, we generated individual co-expression vectors with Cluc and the respective NTCP ortholog to transfect HepG2 cells. At 72 hours post-transfection, NTCP ortholog expression was analyzed indirectly by the secreted Cluc (Fig. 2G). Next, we quantified the [^3^H]-taurocholate uptake (Fig. 2H) and found that all NTCP orthologs maintained the capacity to take up bile acids, although at different efficiencies, probably due to different transfection and expression levels.

**Fig 3:**
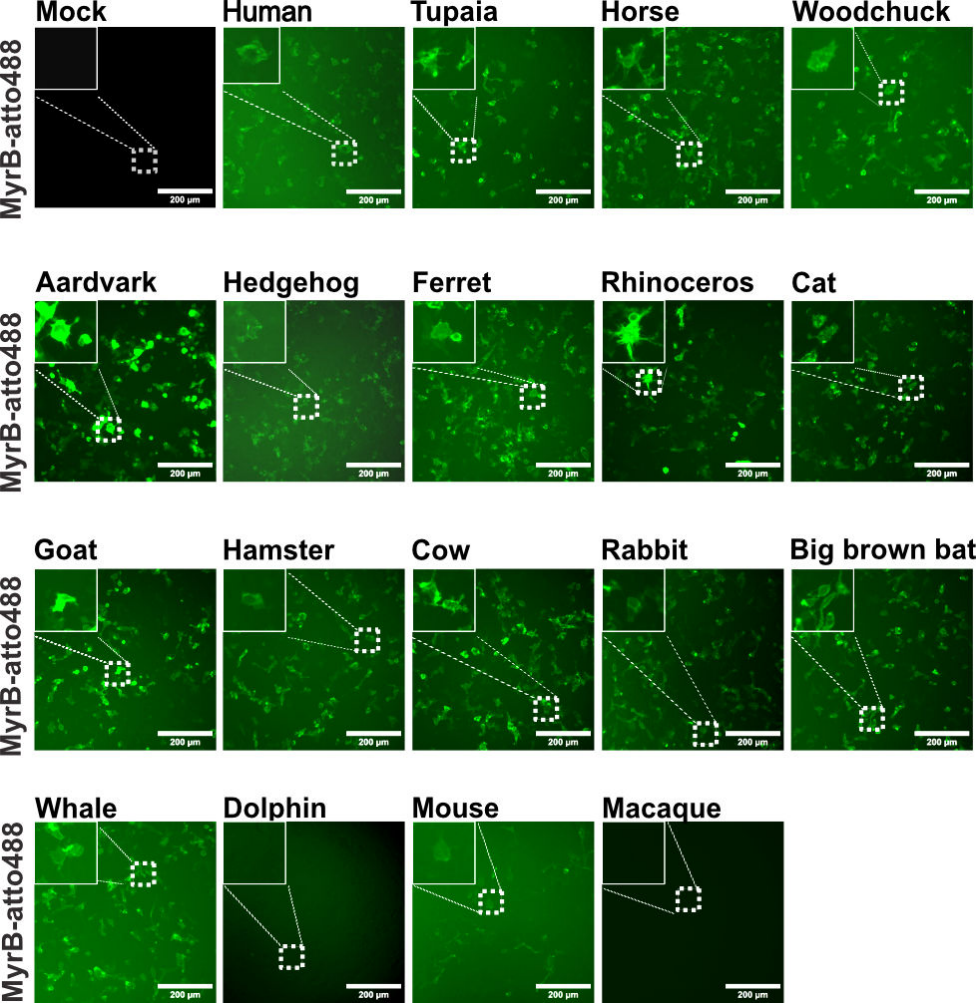
Capability of NTCP orthologs for binding fluorescently labeled MyrB. HepG2 cells were transfected with plasmids expressing the indicated NTCP orthologues. At 3 dpt, cells were incubated with MyrB_atto488_ and analyzed via fluorescence microscopy.

Next, we analyzed the capabilities of the different NTCP orthologs to support HBV PreS1 binding. As before, the HBV binding assay was performed by transfecting HepG2 cells with the expression vectors, followed by MyrB_atto488_ staining. As shown in Fig. 3, MyrB_atto488_ binding was observed in the cells expressing the NTCP orthologs from humans, tupaias, horses, woodchucks, aardvarks, hedgehogs, ferrets, rhinoceroses, cats, goats, hamsters, cows, rabbits, big brown bats, whales, and mice but at different intensities. Cells expressing no NTCP or the orthologs from macaque or dolphin did not show MyrB_atto488_ binding.

**Fig 4:**
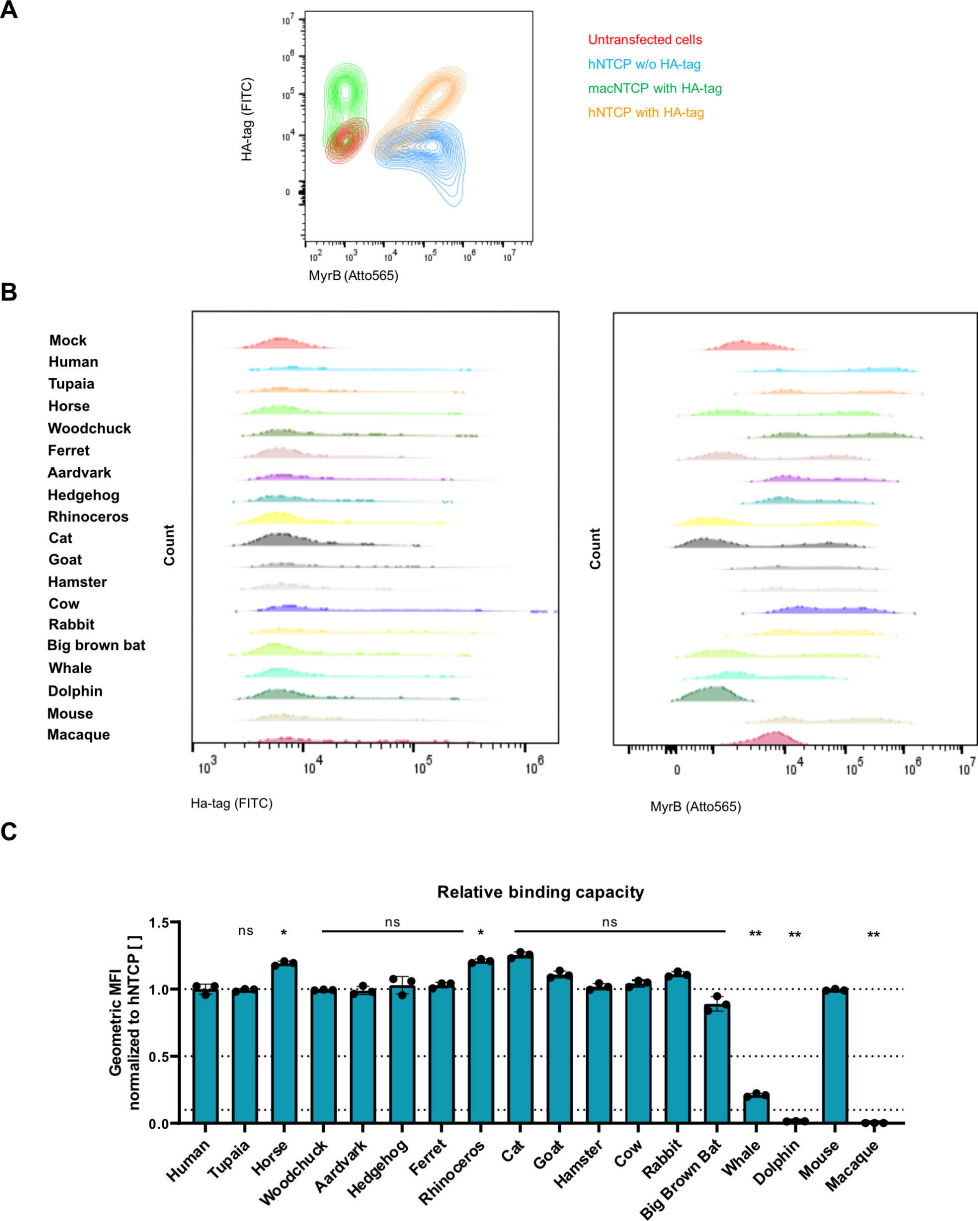
Identification of NTCP orthologs supporting HBV binding. HepG2 cells were transfected with IVT mRNA encoding for the indicated NTCP ortholog fused to an HA-tag. At 24 hours post-transfection, staining was performed using MyrB_atto565_ and anti-HA_alexa488_. (**A**) Flow cytometry analysis of the HepG2 cells transfected with mRNA encoding for hNTCP without an HA-tag (blue), hNTCP with an HA-tag (orange), macaque NTCP (green) with an HA-tag, or nontransfected (red) served as controls. (**B**) Histogram graphs obtained from flow cytometry analysis of HA-NTCP surface expression and MyrB_Atto565_ binding. (**C**) Relative binding capacities of the NTCP orthologs. The geometric mean fluorescence intensity (MFI) for MyrB_atto565_ was calculated for the anti-HA_alexa488_-positive cells. Results were normalized to the geometric MFI of hNTCP. Cut-off lines were drawn at Y = 0.1/0.5/1.0 to define the different groups of NTCP binding ability. Data were compared to those of hNTCP by one-way ANOVA with Dunnett’s multiple comparison correction. Statistical significance is denoted as follows: ****, *P* < 0.0001; **, *P* < 0.01; *, *P* < 0.05; ns, not significant. All experiments were performed in biological triplicates; mean values +/– SD are given.

To further characterize this PreS1-specific binding, we used an *in vitro* transcribed (IVT) mRNA approach to express the NTCP orthologs in high levels (25, 26). To allow the direct quantification of NTCP expression, we fused a hemagglutinin (HA)-tag to the N-terminus of each NTCP ortholog. HepG2 cells were transfected with IVT mRNA and co-stained with an AlexaFluor488-labeled anti-HA-tag antibody and an Atto565-labeled MyrB (MyrB_atto565_) (Fig. 4A). We analyzed the cells via flow cytometry and showed that nontransfected cells neither bound the anti-HA-tag antibody nor the MyrB_atto565_. Cells transfected with hNTCP mRNA, without an HA-tag, bound only MyrB_atto565_, while cells transfected with HA-hNTCP mRNA bound to both the anti-HA-tag antibody and MyrB_atto565_, confirming the feasibility of dual staining. Next, we transfected cells with mRNA of HA-tagged macaque NTCP (HA-macNTCP), known to not bind fluorescently labeled MyrB (3). As expected, we could only detect the binding of the anti-HA-tag antibody via flow cytometry. After successfully validating this approach, we analyzed all NTCP orthologs for their affinity to MyrB_atto565_ in relation to the anti-HA-tag antibody. For this, the gating strategy was adjusted to include only anti-HA-tag positive cells, and the binding of MyrB_atto565_ was analyzed. Surface localization for all NTCP orthologs on IVT mRNA-transfected HepG2 cells (Fig. 4B) could be confirmed through anti-HA-tag staining but at different levels (Fig. S1A). However, MyrB_atto565_ binding was not detected on all NTCPs as dolphin and macaque NTCP-expressing cells did not show MyrB_atto565_ staining (Fig. 4B; Fig. S1B). Affinity to MyrB_atto565_ was normalized to hNTCP and is shown in Fig. 4C. Compared to hNTCP, most NTCP orthologs showed a comparable affinity to that of MyrB_atto565_, except for a higher affinity of horse and rhinoceros NTCP variants and a lower affinity of whale NTCP.

**Fig 5:**
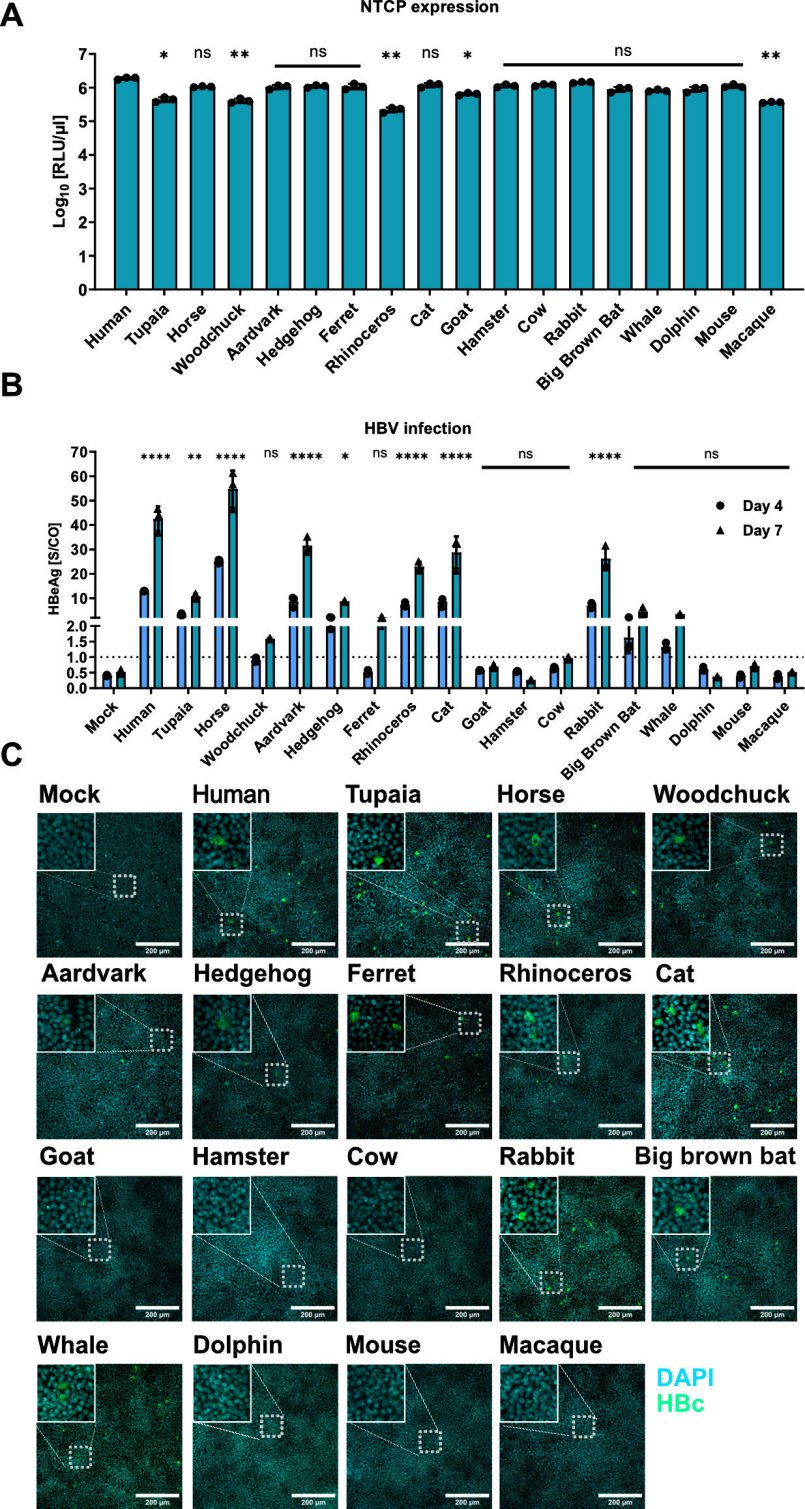
Identification of NTCP orthologs supporting HBV infection. HepG2 cells were transfected with plasmids encoding the different NTCP orthologs and inoculated with HBV. (**A**) Indirect quantification of the NTCP orthologs via Cluc activity. hNTCP served as the positive control. The Kruskal–Wallis test with Dunn’s correction for multiple comparisons was applied. (**B**) HBeAg was measured in the supernatant collected at 4 and 7 dpi; the dotted line indicates the detection limit. Cells transfected with a control plasmid served as a background control, and noninfected cells served as a negative control to set the detection limit. (**C**) Cells were fixed and stained for HBV core protein. (**A**) Data were compared to those of hNTCP, and (**B**) data for 7 dpi were compared to those of the negative control by one-way ANOVA with Dunnett’s correction for multiple comparisons. Statistical significance is denoted as follows: ****, *P* < 0.0001; **, *P* < 0.01; *, *P* < 0.05; ns, not significant. All experiments were performed in biological triplicates; mean values +/– SD are given.

In the next step, we analyzed the NTCP orthologs to determine whether they support HBV infection. We transfected HepG2 cells with the different Cluc/NTCP co-expression plasmids and determined the Cluc activity at 3 days post-transfection (dpt) to ensure a high expression of the NTCP ortholog (Fig. 5A). We then inoculated the cells with HBV and performed HBeAg measurement at 4 and 7 dpi and HBc protein immunostaining at 7 dpi. NTCP orthologs from tupaias, horses, woodchucks, aardvarks, hedgehogs, ferrets, rhinoceroses, cats, rabbits, big brown bats, and whales showed HBeAg expression above the threshold at different levels (Fig. 5B) and detectable intracellular HBc protein (Fig. 5C), indicating that they support HBV infection. As expected, mouse and macaque NTCP did not mediate HBV infection. Dolphin NTCP, which did not bind HBV PreS1 in the previous experiments, did not mediate HBV infection, probably due to the described G158N mutation (12). However, the NTCP orthologs from hamsters, goats, and cows that were capable of binding HBV PreS1 also did not mediate HBV infection.

### Modification of NTCP orthologs at the functional domain or in proximity allows HBV infection mediated by chimeric hamster, goat, and cow NTCP variants

**Fig 6:**
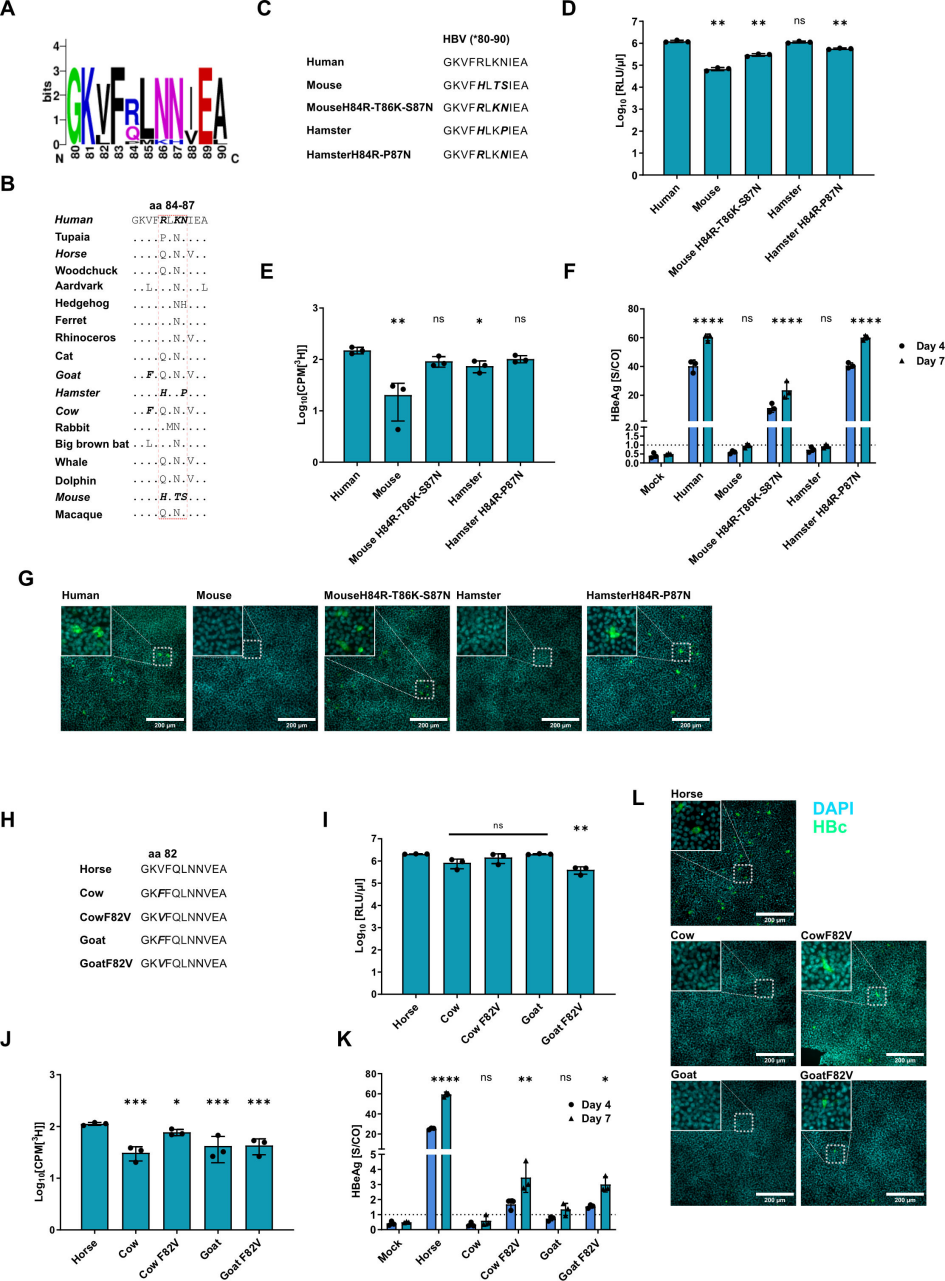
Modification of NTCP orthologs to support HBV infection. (**A**) Sequence conservation analysis among the HBV permissive NTCP orthologs. (**B**) Sequence alignment of the NTCP functional domains (aa 84–87) compared to hNTCP as a reference. (**C, H**) Amino acid alignment of the different NTCP orthologs and chimeric variants. (**D–G, I–L**) HepG2 cells were transfected to express the chimeric NTCP orthologs, and the expression levels (**D, I**), the bile acid transporter capability (**E, J**), and HBeAg (**F, G**) were quantified. Intracellular HBV core protein (**K, L**) was stained. (**F, K**) The dotted lines indicate the cut-off between nonreactive and reactive. All experiments were performed in biological triplicates; mean values +/– SD are given. Data were compared to those of human (**D, E**) or horse NTCP (**I, J**) by one-way ANOVA with Dunnett’s multiple comparison correction. (**F, K**) Data for 7 dpi were compared to those of the negative control by one-way ANOVA with Dunnett’s correction for multiple comparisons. Statistical significance is denoted as follows: ****, *P* < 0.0001; ***, *P* < 0.001; **, *P* < 0.01; *, *P* < 0.05; ns, not significant.

To further investigate why hamster, goat, and cow NTCP variants could not mediate HBV infection despite being able to bind HBV PreS1, we compared the amino acid sequences in proximity to the previously described functional domain at aa 84–87. As shown in Fig. 6A, aa variations 84 (R or Q), 86 (K or N), and 87 (N or H) are present in HBV-permissive NTCP variants. While cow and goat NTCP variants showed high similarities in that region, we noted H84 and P87 in hamster NTCP (Fig. 6B). Thus, we exchanged the two amino acids of hamster NTCP (H84R and P87N) and analyzed the chimeric NTCP variant expression as well as its physiological function for bile acid uptake. We included murine NTCP and the previously described chimeric mouse/human NTCP variant (H84R/T86K/S87N) as controls. Indeed, we could confirm a functionally expressed chimeric hamster/human NTCP (H84R and P87N) variant (Fig. 6C through E). In the next step, we tested the chimeric hamster/human NTCP variant for its capability of supporting HBV infection. We transfected HepG2 cells with the different NTCP variants and inoculated the cells with HBV at 3 dpt. As before, hNTCP served as a positive control, and nontransfected HepG2 cells served as a negative control. HBeAg expression levels at 4 and 7 dpi (Fig. 6F) and detectable intracellular HBc protein (Fig. 6G) revealed that the expression of the chimeric hamster/human NTCP variants supported HBV infection.

Next, we analyzed the sequences of cow and goat NTCP variants. While both orthologs harbor aa variations Q84 and N86, like the functional NTCP ortholog from horses, we noticed an additional difference in amino acids in goat and cow NTCP at position 82 (F82 instead of V82) (Fig. 6B). To determine if this exchange at residue 82 impedes HBV infection, we replaced aa 82 in goat and cow NTCP with its human counterpart (exchange F82V) and subsequently evaluated the chimeric NTCP variant for expression, functionality for bile acid uptake, and HBV infection as before (Fig. 6H through L). Horse NTCP served as a positive control, supporting HBV infection (Fig. 5B and C) and harboring the aa Q84 and N86. The chimeric NTCP variants were expressed at a comparable level and maintained their physiological functionality as bile acid transporters (Fig. 6I and J). Furthermore, low levels of HBeAg above the threshold at 4 and 7 dpi (Fig. 6K) and intracellular HBc protein (Fig. 6L) were detected, indicating that goat and cow NTCP F82V can support HBV infection. In summary, this shows that the F82V exchange rendered chimeric goat and cow NTCP variants functional for HBV binding and entry. However, mutation of horse NTCP with V82F only reduced HBeAg and intracellular HBV cccDNA (Fig. S2), but did not abolish horse NTCP functionality, which additionally suggests that other amino acids in close proximity to the previously described functional domain of NTCP (aa 84–87) might be relevant for HBV infection.

### Functionality of primary hepatocytes for HBV uptake and infection

**Fig 7:**
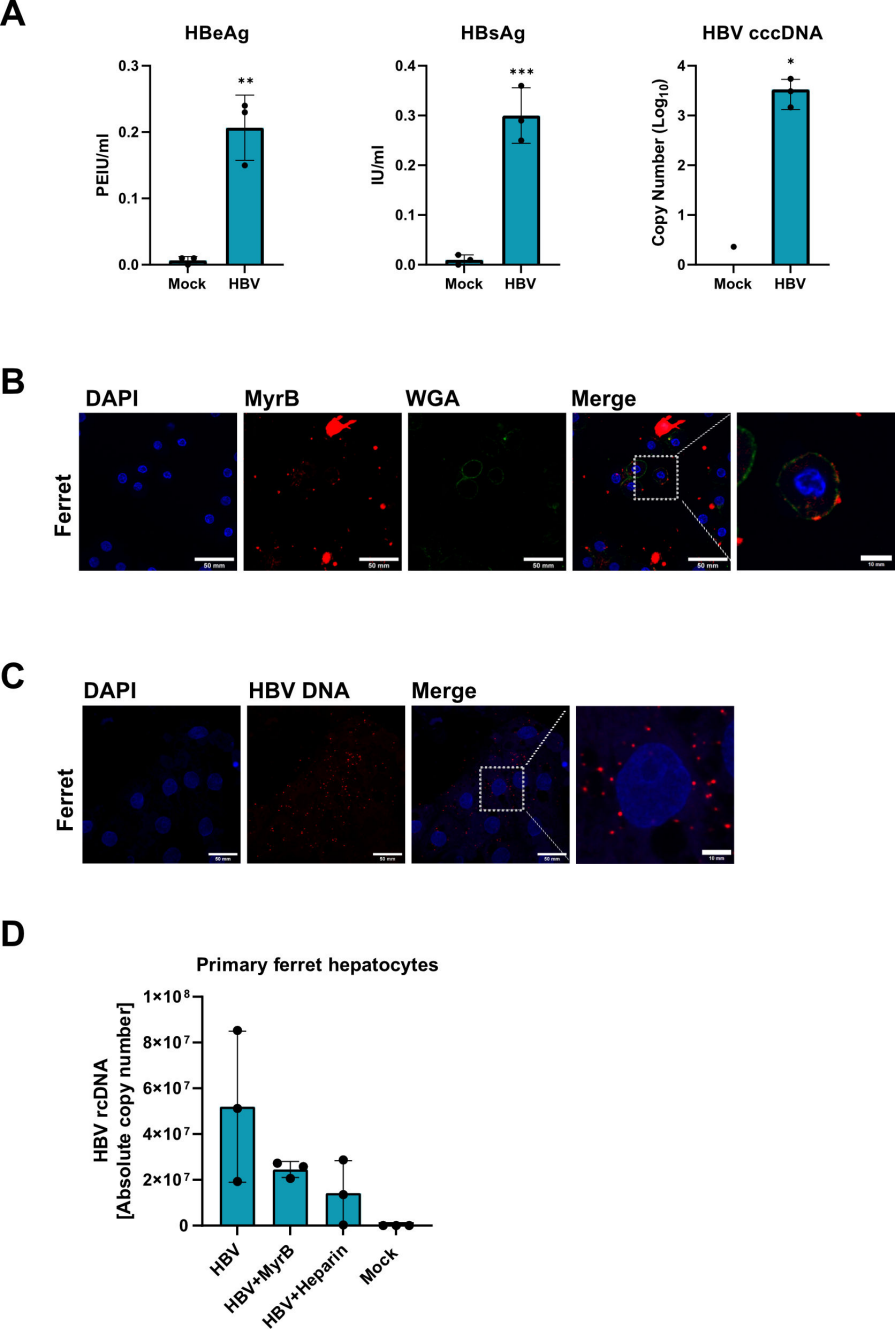
Evaluation of primary hepatocytes for HBV uptake and infection. (**A**) Primary horse hepatocytes were plated and inoculated with HBV. At 7 dpi, HBeAg, HBsAg, and cccDNA were measured. (**B, C, D**) Primary ferret hepatocytes were isolated and seeded. (**B**) One day post-seeding, the cells were co-stained by MyrB_atto565_ and WGA_Alex488_. (C + D) A synchronized infection was performed with the hepatocytes and cells analyzed for intracellular HBV DNA via (**C**) RNAscope or (**D**) qPCR. Data were compared to those of the negative control by one-way ANOVA with Dunnett’s correction for multiple comparisons. Statistical significance is denoted as follows: ***, *P* < 0.001; **, *P* < 0.01; *, *P* < 0.05; ns, not significant. WGA, wheat germ agglutinin.

To determine whether our results are applicable to a potential permissiveness of the species-specific hepatocytes, we reviewed the literature and found a previous report suggesting that primary horse hepatocytes (PHoH) are permissive to HBV infection (19). Considering this result, we purchased cryopreserved PHoH and performed an HBV infection assay. At 7 dpi, we detected HBeAg, HBsAg, and intracellular HBV cccDNA in PHoH (Fig. 7A), confirming that these cells are fully permissive to HBV.

To further validate our results in other species-specific hepatocytes, we isolated primary ferret hepatocytes (PFH) and co-stained the hepatocytes with MyrB_atto565_ and, to highlight the membranes, with WGA_alexa488_. As shown in Fig. 7B, MyrB_atto565_ binding was evident on the membranes of PFH. We next inoculated the cells with HBV to assess their permissiveness and quantified HBeAg, HBsAg, and HBV cccDNA at 7 dpi. However, all HBV infection parameters were negative (data not shown), indicating that PFH do not support HBV infection. To determine whether HBV enters the cells, we inoculated PFH with HBV and performed qPCR and an *in situ* hybridization RNAscope assay to detect intracellular HBV rcDNA. Intracellular HBV rcDNA was detectable in PFH by both the RNAscope assay (Fig. 7C) and qPCR (Fig. 7D), indicating a block in HBV infection at a post-entry step in PFH.

## DISCUSSION

The establishment of new HBV animal models is hindered by the strict species tropism of the virus (11). The discovery of NTCP as the bona fide entry receptor has identified a key factor for HBV species specificity (3). In our study, we selected 12 NTCP orthologs based on their similarity with hNTCP aa 157–165 and evaluated their capability to render HepG2 cells susceptible to HBV. Our data show that nonhuman NTCP orthologs from woodchucks, ferrets, aardvarks, horses, rabbits, whales, big brown bats, cats, and rhinoceroses support HBV binding and entry. These results help identify species that may be capable of supporting *de novo* HBV infection and thus may serve as potential HBV animal models. Moreover, these species could be potential hosts for yet unknown HBV reservoirs. However, they need to be screened for their ability to support critical post-entry steps of HBV infection.

A cat hepadnavirus has been identified by several groups (27–31), which uses the feline NTCP as a major cell entry receptor (32). These findings confirm our result that cat NTCP supports HBV infection. While Shofa et al. showed that a PreS1 peptide derived from HBV or the cat hepadnavirus could bind to several species-specific NTCP orthologs, including cat and cow NTCP (31), we demonstrated that HBV PreS1 binding of cow, hamster, and goat NTCP does not necessarily imply these NTCP orthologs support HBV infection. Moreover, we showed that the previously described functional domain of NTCP at aa 84–87 (15), as well as amino acid residues in close proximity to it, are essential for HBV infection in the NTCP orthologs we investigated. This is in line with a recent report showing that the humanization of hamster NTCP at aa H84R/P87N is sufficient to support HBV infection (33).

Using cryo-electron microscopy, recent reports unveiled the structure of NTCP and mapped the interaction of aa residues between NTCP and the HBV PreS1 peptide (34–38). Interestingly, the functional domain of NTCP at aa 84–87 is located at the TM2–TM3 loop, outside of the extracellular surface region. While the N-terminal part of the HBV PreS1 peptide binds directly inside the NTCP tunnel, the C-terminal part, especially D43 and W41, interacts with aa N87 of hNTCP. Variations at this position or in its close proximity, as indicated by our results, might change the folding of the aa 87 side chain, therefore limiting the interaction between NTCP and HBV without affecting the bile acid uptake. Subsequently, studies like ours are necessary to individually analyze whether a species-specific NTCP ortholog supports HBV infection. However, it has been shown that the expression of an NTCP variant supporting HBV infection does not necessarily result in HBV permissiveness of the species' hepatocytes (15, 17). As shown in mice, post-entry steps like the nuclear import of the HBV capsid might restrict HBV infection (18). Our results indicate that this might also be the case in PFH where HBV entry, but no infection, could be detected. Thus, further experiments like the isolation and challenge of species-specific primary hepatocytes, especially from cats and rabbits, might be necessary to shortlist these species as potential animal models.

In summary, we established a set of methods to investigate species-specific NTCP orthologs and chimeras for their functionality in supporting HBV binding and infection and identified NTCP orthologs from ferrets, aardvarks, horses, rabbits, whales, big brown bats, and rhinoceroses as potential HBV-entry receptors. Our results indicate that species like cats and rabbits should be investigated with respect to their potential to serve as new HBV infection animal models.

## MATERIALS AND METHODS

### Cell culture

Human hepatocellular carcinoma cells (HepG2) (ATCCHB-8065) and HepG2-NTCP-K7 (8) cell lines were cultured in Dulbecco’s modified Eagle medium (Gibco, Thermo Fischer Scientific) supplemented with 10% heat-inactivated fetal calf serum (FCS; Gibco, Thermo Fischer Scientific) and 1% penicillin/streptomycin (10.000 U/mL, Gibco, Thermo Fischer Scientific), 2 mM glutamine (Gibco, Thermo Fischer Scientific), 1 × nonessential amino acids (Gibco, Thermo Fischer Scientific), and 1 mM sodium pyruvate (Gibco, Thermo Fischer Scientific) in a humidified 37°C, 5% CO_2_ incubator with regular passage every 4 days. To differentiate HepG2 and HepG2-NTCP-K7 cells, 2.5% DMSO was added to the cell culture medium for 3 days after seeding. HepG2 and HepG2-NTCP-K7 cells were cultivated on collagen-coated plates. All experiments were performed in 24-well plates using 500 µL of standard cultivation or infection medium per well.

### Cloning and mRNA production

NTCP sequences were synthesized via GeneArt Strings DNA Fragments service (Invitrogen, Thermo Fisher Scientific). The DNA fragments were amplified by PCR with primers in Table S1 and inserted into the pcDNA3.1-Cluc backbone under the control of a CMV promoter. For chimeric-NTCP, an additional PCR was performed to get two separated DNA sequences that contain a 15–20 bp overlap sequence with target mutation. Then, a second round of PCR with a mixture of two separated DNA sequences as the template was performed, and the chimeric-NTCP fragments were inserted into the pcDNA3.1-Cluc backbone. For the mRNA production, NTCP variant sequences were amplified and inserted into the pcDNA3.1 backbone under the control of the T7 promoter. Sequences of all NTCP plasmids were confirmed by the DNA Sanger sequence service. IVT mRNA production was performed using the HiScribe ARCA T7 *in vitro* transcription kit (New England Biolabs, Ipswich, MA, USA) supplemented with 10 µM ψ-UTP and 10 µM m5 CTP nucleotide analogs (Jena Bioscience, Jena, Germany) as indicated according to the previous description (25). Synthesized IVT mRNA was analyzed on 2% agarose gel to validate tailing efficiency and correct length.

### NTCP expression quantification

Supernatants from transfected cells and nontransfected cells were collected at 3 dpt and measured at 1:1,000 dilution using the Cypridina luciferase detection kit (New England Biolabs, Ipswich, MA, USA) with Tecan reader infinite 200 Pro (TECAN, Männedorf, Swizerland). Supernatants from nontransfected cells served as a negative control.

### MyrB binding assay

MyrB binding assay was performed as previously described (8). In brief, HepG2 cells were transfected with plasmids expressing NTCP variants and incubated with 200 nM MyrB_atto488_ or MyrB_atto565_ (LifeTein, Franklin Township, USA) in the culture medium at 37°C for 30 minutes at 3 dpt. After washing twice with PBS, the fluorescence images were captured with a Leica fluorescence microscope (LAS X, Leica, Wetzlar, Germany). Primary hepatocytes were counterstained with the plasma membrane marker WGA coupled to Alexa-Fluor 488 (Thermo Fisher Scientific) diluted 1:500 for 30 minutes.

### Flow cytometry

HepG2 cells were seeded and transfected with NTCP variant IVT mRNAs. The cells were trypsinized at 24 hours post-transfection and co-stained with MyrB_atto565_ at 200 nM and anti-HA_alexa488_ (1:1000; Invitrogen) for 30 minutes. After washing twice with FACS buffer, the cell pellets were suspended with 200 µL FACS buffer per well and then analyzed by flow cytometry (Cytoflex, Beckman Coulter, Brea, CA, USA). Data processing was performed using FlowJo software (Becton Dickinson).

### Taurocholate uptake assay

[^3^H] taurocholate uptake assay for all NTCP variants expressing cells was performed as previously described (39). Briefly, Hot Mix stock ((1,940 µL basal medium, 66 µL 15 mM cold TC (Sigma-Aldrich), and 1 µL hot TC (Hartmann Analytic, Braunschweig, Germany)) was prepared and diluted 1:10 in the basal medium, and 250 µL/well of Hot Mix was added to the samples and incubated for 15 minutes at 37°C. Cells were placed on ice after incubation and washed three times with ice-cold PBS. Next, cells were lysed with 500 µL lysis buffer (0.05% SDS, 0.25 mM NaOH), and the cell lysate was transferred to scintillation vials. Finally, vials were closed after adding 4 mL scintillation liquid and vortexed for 30 seconds. Measurement of [^3^H] taurocholate was performed with a scintillation analyzer.

### HBV infection assay

Cells were cultured in standard cultivation medium containing 2.5% DMSO for 2 dpt and then inoculated with 1,000 multiplicities of infection (MOI) viral particles/cell at 37°C for 24 hours in the presence of 5% polyethylene glycol (PEG). HBV particles were produced by HepAD38 cells as described (40). The supernatant from infected cells expressing NTCP variants was collected at 4 and 7 dpi. Secreted HBeAg was quantitatively measured using a commercial immunoassay kit (BEP III, Siemens Molecular Diagnostics, Marburg, Germany) with HBeAg Quantitative Calibrators on the Architect platform (Abbott Laboratories, Chicago, IL, USA). Sample/cut-off was determined using the internal cut-off value, whereas samples > 1 are considered positive.

### Imaging of HBV core protein

HBV core protein of infected cells was immunostained as previously described (8). Briefly, HBV-infected cells seeded on 12 mm coverslips were fixed with 4% paraformaldehyde and permeabilized with 0.5% saponin. Cells were then incubated with rabbit anti-core serum (Cell Marque), followed by Alexa Flour 488-coupled secondary antibody incubation (Invitrogen, Carlsbad, CA, USA). After immunostaining, coverslips were mounted with Flouromount-G containing DAPI (SouthernBiotech, Birmingham, AL, USA), and images were collected by using a Leica DMi8 fluorescence microscope (Leica, Wetzlar, Germany).

### Primary hepatocyte isolation

Primary hepatocytes were isolated as previously described with minimal media adjustments (41). In brief, the livers were dissected into liver lobes, followed by individual flushing by perfusion buffer containing 1 x Lefferts (10 HEPES, 2 mM KCl, 10 Mm NaCl, and 1.2 mM NaH_2_PO_4_-H_2_O) and 1 x EGTA (5 mM EGTA Solution in 1 x Lefferts Buffer) for about 10 minutes. Subsequently, the lobes were digested by rinsing with a digestion buffer (1 x Lefferts, 2 mM CaCl2, and collagenase type 4) for 20–30 minutes. The capsule was then opened, and cells were removed from the tissue with a cell scraper and centrifuged for 5 minutes at 4°C at 50 g. After multiple rounds of centrifugation and washing, the cells were seeded on collagenated plates.

### RNAscope assay

Isolated primary hepatocytes were seeded on 12 mm coverslips and inoculated with wtHBV. After 16 hours, cells were fixed with 4% paraformaldehyde and pretreated with hydrogen peroxide and protease at RT for 10 minutes, respectively. The RNAscope assay was performed with the RNAscope Multiplex Fluorescent Detection Kit v2 (323100, ACDbio, CA). Intracellular rcDNA was detected by the RNAscope Probe-V-HBV-awy-sense (511681, ACDbio, CA), which targets the HBV S region (GenBank: U95551.1). The images were captured by using Zeiss LSM 900 Airyscan 2 confocal microscope (Zeiss, Jena, Germany).

### HBV sequence analysis

The amino acid sequences of HBV functional and binding domains were aligned by Genedoc 2.7, and conserved analysis was created by WebLogo 2.8.2.

### Statistics

GraphPad Prism Version 10.0.2 by Graphpad Software Inc., San Diego, California, USA, was used to statistically analyze and plot the results.

## SUPPLEMENTAL MATERIAL

Supplemental material jvi.01833-24-s0001.pdf Data regarding NTCP surface expression, MyrB binding, and infection for horse NTCP V82F.

ASM does not own the copyrights to Supplemental Material that may be linked to, or accessed through, an article. The authors have granted ASM a non-exclusive, world-wide license to publish the Supplemental Material files. Please contact the corresponding author directly for reuse.

## References

[1] WHO. 2024. Fact Sheets - Hepatitis B. Available from: https://www.who.int/news-room/fact-sheets/detail/hepatitis-b

[2] Schulze A, Gripon P, Urban S. 2007. Hepatitis B virus infection initiates with a large surface protein-dependent binding to heparan sulfate proteoglycans. Hepatology 46:1759–1768. doi:10.1002/hep.21896 18046710

[3] Yan H, Zhong G, Xu G, He W, Jing Z, Gao Z, Huang Y, Qi Y, Peng B, Wang H, Fu L, Song M, Chen P, Gao W, Ren B, Sun Y, Cai T, Feng X, Sui J, Li W. 2012. Sodium taurocholate cotransporting polypeptide is a functional receptor for human hepatitis B and D virus. Elife 3:e00049. doi:10.7554/eLife.00049 PMC3485615 23150796

[4] Ni Y, Lempp FA, Mehrle S, Nkongolo S, Kaufman C, Fälth M, Stindt J, Königer C, Nassal M, Kubitz R, Sültmann H, Urban S. 2014. Hepatitis B and D viruses exploit sodium taurocholate co-transporting polypeptide for species-specific entry into hepatocytes. Gastroenterology 146:1070–1083. doi:10.1053/j.gastro.2013.12.024 24361467

[5] Appelman MD, Wettengel JM, Protzer U, Oude Elferink RPJ, van de Graaf SFJ. 2021. Molecular regulation of the hepatic bile acid uptake transporter and HBV entry receptor NTCP. Biochim Biophys Acta Mol Cell Biol Lipids 1866:158960. doi:10.1016/j.bbalip.2021.158960 33932583

[6] Iwamoto M, Watashi K, Tsukuda S, Aly HH, Fukasawa M, Fujimoto A, Suzuki R, Aizaki H, Ito T, Koiwai O, Kusuhara H, Wakita T. 2014. Evaluation and identification of hepatitis B virus entry inhibitors using HepG2 cells overexpressing a membrane transporter NTCP. Biochem Biophys Res Commun 443:808–813. doi:10.1016/j.bbrc.2013.12.052 24342612

[7] Michailidis E, Pabon J, Xiang K, Park P, Ramanan V, Hoffmann H-H, Schneider WM, Bhatia SN, de Jong YP, Shlomai A, Rice CM. 2017. A robust cell culture system supporting the complete life cycle of hepatitis B virus. Sci Rep 7:16616. doi:10.1038/s41598-017-16882-5 29192196 PMC5709435

[8] Ko C, Chakraborty A, Chou W-M, Hasreiter J, Wettengel JM, Stadler D, Bester R, Asen T, Zhang K, Wisskirchen K, McKeating JA, Ryu W-S, Protzer U. 2018. Hepatitis B virus genome recycling and de novo secondary infection events maintain stable cccDNA levels. J Hepatol 69:1231–1241. doi:10.1016/j.jhep.2018.08.012 30142426 PMC7611400

[9] Seeger C, Sohn JA. 2014. Targeting hepatitis B virus with CRISPR/Cas9. Mol Ther Nucleic Acids 3:e216. doi:10.1038/mtna.2014.68 25514649 PMC4272409

[10] Shimura S, Watashi K, Fukano K, Peel M, Sluder A, Kawai F, Iwamoto M, Tsukuda S, Takeuchi JS, Miyake T, Sugiyama M, Ogasawara Y, Park S-Y, Tanaka Y, Kusuhara H, Mizokami M, Sureau C, Wakita T. 2017. Cyclosporin derivatives inhibit hepatitis B virus entry without interfering with NTCP transporter activity. J Hepatol 66:685–692. doi:10.1016/j.jhep.2016.11.009 27890789 PMC7172969

[11] Wettengel JM, Burwitz BJ. 2020. Innovative HBV animal models based on the entry receptor NTCP. Viruses 12:828. doi:10.3390/v12080828 32751581 PMC7472226

[12] Müller SF, König A, Döring B, Glebe D, Geyer J. 2018. Characterisation of the hepatitis B virus cross-species transmission pattern via Na^+^/taurocholate co-transporting polypeptides from 11 New World and Old World primate species. PLoS ONE 13:e0199200. doi:10.1371/journal.pone.0199200 29912972 PMC6005513

[13] Lempp FA, Wiedtke E, Qu B, Roques P, Chemin I, Vondran FWR, Le Grand R, Grimm D, Urban S. 2017. Sodium taurocholate cotransporting polypeptide is the limiting host factor of hepatitis B virus infection in macaque and pig hepatocytes. Hepatology 66:703–716. doi:10.1002/hep.29112 28195359

[14] Burwitz BJ, Wettengel JM, Mück-Häusl MA, Ringelhan M, Ko C, Festag MM, Hammond KB, Northrup M, Bimber BN, Jacob T, et al.. 2017. Hepatocytic expression of human sodium-taurocholate cotransporting polypeptide enables hepatitis B virus infection of macaques. Nat Commun 8:2146. doi:10.1038/s41467-017-01953-y 29247188 PMC5732258

[15] Yan H, Peng B, He W, Zhong G, Qi Y, Ren B, Gao Z, Jing Z, Song M, Xu G, Sui J, Li W. 2013. Molecular determinants of hepatitis B and D virus entry restriction in mouse sodium taurocholate cotransporting polypeptide. J Virol 87:7977–7991. doi:10.1128/JVI.03540-12 23678176 PMC3700185

[16] He W, Cao Z, Mao F, Ren B, Li Y, Li D, Li H, Peng B, Yan H, Qi Y, Sun Y, Wang F, Sui J, Li W. 2016. Modification of three amino acids in sodium taurocholate cotransporting polypeptide renders mice susceptible to infection with hepatitis D virus in vivo. J Virol 90:8866–8874. doi:10.1128/JVI.00901-16 27466423 PMC5021397

[17] Li H, Zhuang Q, Wang Y, Zhang T, Zhao J, Zhang Y, Zhang J, Lin Y, Yuan Q, Xia N, Han J. 2014. HBV life cycle is restricted in mouse hepatocytes expressing human NTCP. Cell Mol Immunol 11:175–183. doi:10.1038/cmi.2013.66 24509445 PMC4003384

[18] Zhao K, Guo F, Wang J, Zhong Y, Yi J, Teng Y, Xu Z, Zhao L, Li A, Wang Z, Chen X, Cheng X, Xia Y. 2023. Limited disassembly of cytoplasmic hepatitis B virus nucleocapsids restricts viral infection in murine hepatic cells. Hepatology 77:1366–1381. doi:10.1002/hep.32622 35718932

[19] Rasche A, Lehmann F, Goldmann N, Nagel M, Moreira-Soto A, Nobach D, de Oliveira Carneiro I, Osterrieder N, Greenwood AD, Steinmann E, Lukashev AN, Schuler G, Glebe D, Drexler JF, Equid HBV Consortium. 2021. A hepatitis B virus causes chronic infections in equids worldwide. Proc Natl Acad Sci USA 118:13. doi:10.1073/pnas.2013982118 PMC8020657 33723007

[20] Fu L, Hu H, Liu Y, Jing Z, Li W. 2017. Woodchuck sodium taurocholate cotransporting polypeptide supports low-level hepatitis B and D virus entry. Virology (Auckl) 505:1–11. doi:10.1016/j.virol.2017.02.006 28213271

[21] Chng J, Wang T, Nian R, Lau A, Hoi KM, Ho SCL, Gagnon P, Bi X, Yang Y. 2015. Cleavage efficient 2A peptides for high level monoclonal antibody expression in CHO cells. MAbs 7:403–412. doi:10.1080/19420862.2015.1008351 25621616 PMC4622431

[22] Wettengel JM, Naka H, Dissen GA, Torgerson J, Pounder M, Mueller SF, Mueller E, Hagen P, Brandt M, Protzer U, Burwitz BJ. 2024. High-throughput screening for the prevalence of neutralizing antibodies against human adenovirus serotype 5. Vaccines (Basel) 12:155. doi:10.3390/vaccines12020155 38400138 PMC10891882

[23] Gripon P, Cannie I, Urban S. 2005. Efficient inhibition of hepatitis B virus infection by acylated peptides derived from the large viral surface protein. J Virol 79:1613–1622. doi:10.1128/JVI.79.3.1613-1622.2005 15650187 PMC544121

[24] Zhong G, Yan H, Wang H, He W, Jing Z, Qi Y, Fu L, Gao Z, Huang Y, Xu G, Feng X, Sui J, Li W. 2013. Sodium taurocholate cotransporting polypeptide mediates woolly monkey hepatitis B virus infection of Tupaia hepatocytes. J Virol 87:7176–7184. doi:10.1128/JVI.03533-12 23596296 PMC3676132

[25] Oswald A, Chakraborty A, Ni Y, Wettengel JM, Urban S, Protzer U. 2021. Concentration of Na^+^-taurocholate-cotransporting polypeptide expressed after in vitro-transcribed mRNA transfection determines susceptibility of hepatoma cells for hepatitis B virus. Sci Rep 11:19799. doi:10.1038/s41598-021-99263-3 34611272 PMC8492621

[26] Jeske SD, Wettengel JM, Gegenfurtner F, Fischer K, Moosmüller J, Chakraborty A, Ko C, Burwitz BJ, Schnieke A, Protzer U. 2024. Identification of amino acids restricting HBV receptor function in porcine NTCP. npj Viruses 2:30. doi:10.1038/s44298-024-00041-5 PMC11721346 40295677

[27] Aghazadeh M, Shi M, Barrs VR, McLuckie AJ, Lindsay SA, Jameson B, Hampson B, Holmes EC, Beatty JA. 2018. A novel hepadnavirus identified in an immunocompromised domestic cat in Australia. Viruses 10:269. doi:10.3390/v10050269 29772771 PMC5977262

[28] Jeanes EC, Wegg ML, Mitchell JA, Priestnall SL, Fleming L, Dawson C. 2022. Comparison of the prevalence of Domestic Cat Hepadnavirus in a population of cats with uveitis and in a healthy blood donor cat population in the United Kingdom. Vet Ophthalmol 25:165–172. doi:10.1111/vop.12956 34806802

[29] Stone C, Petch R, Gagne RB, Nehring M, Tu T, Beatty JA, VandeWoude S. 2022. Prevalence and genomic sequence analysis of domestic cat hepadnavirus in the United States. Viruses 14:2091. doi:10.3390/v14102091 36298647 PMC9607532

[30] Capozza P, Carrai M, Choi YR, Tu T, Nekouei O, Lanave G, Martella V, Beatty JA, Barrs VR. 2023. Domestic cat hepadnavirus: molecular epidemiology and phylogeny in cats in Hong Kong. Viruses 15:150. doi:10.3390/v15010150 36680190 PMC9865086

[31] Takahashi K, Kaneko Y, Shibanai A, Yamamoto S, Katagiri A, Osuga T, Inoue Y, Kuroda K, Tanabe M, Okabayashi T, Naganobu K, Minobe I, Saito A. 2022. Identification of domestic cat hepadnavirus from a cat blood sample in Japan. J Vet Med Sci 84:648–652. doi:10.1292/jvms.22-0010 35321970 PMC9177394

[32] Shofa M, Ohkawa A, Kaneko Y, Saito A. 2023. Conserved use of the sodium/bile acid cotransporter (NTCP) as an entry receptor by hepatitis B virus and domestic cat hepadnavirus. Antiviral Res 217:105695. doi:10.1016/j.antiviral.2023.105695 37536428

[33] Zhang H, Liu Y, Liu C-D, Wang Z, Guo H. 2024. The feasibility of establishing a hamster model for HBV infection: in vitro evidence. MBio 15:e0261524. doi:10.1128/mbio.02615-24 39329526 PMC11559161

[34] Park J-H, Iwamoto M, Yun J-H, Uchikubo-Kamo T, Son D, Jin Z, Yoshida H, Ohki M, Ishimoto N, Mizutani K, Oshima M, Muramatsu M, Wakita T, Shirouzu M, Liu K, Uemura T, Nomura N, Iwata S, Watashi K, Tame JRH, Nishizawa T, Lee W, Park S-Y. 2022. Structural insights into the HBV receptor and bile acid transporter NTCP. Nature 606:1027–1031. doi:10.1038/s41586-022-04857-0 35580630 PMC9242859

[35] Asami J, Kimura KT, Fujita-Fujiharu Y, Ishida H, Zhang Z, Nomura Y, Liu K, Uemura T, Sato Y, Ono M, Yamamoto M, Noda T, Shigematsu H, Drew D, Iwata S, Shimizu T, Nomura N, Ohto U. 2022. Structure of the bile acid transporter and HBV receptor NTCP. Nature 606:1021–1026. doi:10.1038/s41586-022-04845-4 35580629

[36] Goutam K, Ielasi FS, Pardon E, Steyaert J, Reyes N. 2022. Structural basis of sodium-dependent bile salt uptake into the liver. Nature 606:1015–1020. doi:10.1038/s41586-022-04723-z 35545671 PMC9242856

[37] Asami J, Park J-H, Nomura Y, Kobayashi C, Mifune J, Ishimoto N, Uemura T, Liu K, Sato Y, Zhang Z, Muramatsu M, Wakita T, Drew D, Iwata S, Shimizu T, Watashi K, Park S-Y, Nomura N, Ohto U. 2024. Structural basis of hepatitis B virus receptor binding. Nat Struct Mol Biol 31:447–454. doi:10.1038/s41594-023-01191-5 38233573

[38] Liu H, Zakrzewicz D, Nosol K, Irobalieva RN, Mukherjee S, Bang-Sørensen R, Goldmann N, Kunz S, Rossi L, Kossiakoff AA, Urban S, Glebe D, Geyer J, Locher KP. 2024. Structure of antiviral drug bulevirtide bound to hepatitis B and D virus receptor protein NTCP. Nat Commun 15:2476. doi:10.1038/s41467-024-46706-w 38509088 PMC10954734

[39] Kubitz R, Sütfels G, Kühlkamp T, Kölling R, Häussinger D. 2004. Trafficking of the bile salt export pump from the Golgi to the canalicular membrane is regulated by the p38 MAP kinase. Gastroenterology 126:541–553. doi:10.1053/j.gastro.2003.11.003 14762791

[40] Wettengel JM, Linden B, Esser K, Laue M, Burwitz BJ, Protzer U. 2021. Rapid and robust continuous purification of high-titer hepatitis B virus for in vitro and in vivo applications. Viruses 13:1503. doi:10.3390/v13081503 34452368 PMC8402639

[41] Esser K, Cheng X, Wettengel JM, Lucifora J, Hansen-Palmus L, Austen K, Roca Suarez AA, Heintz S, Testoni B, Nebioglu F, et al.. 2023. Hepatitis B virus targets lipid transport pathways to infect hepatocytes. Cell Mol Gastroenterol Hepatol 16:201–221. doi:10.1016/j.jcmgh.2023.03.011 37054914 PMC10394270

