# Supplementary material for: Identification of NTCP animal orthologs supporting hepatitis B virus binding and infection
Source: J Virol. 2025 Mar 5;99(4):e01833-24. doi: 10.1128/jvi.01833-24 (PMC11998539; doi:10.1128/jvi.01833-24)
Supplement: Supplemental material — Data regarding NTCP surface expression, MyrB binding, and infection for horse NTCP V82F. [file jvi.01833-24-s0001.pdf]

Identification of NTCP animal orthologues supporting hepatitis B virus binding and infection

Fuwang Chen,[1] Jochen M. Wettengel,[1,2] Florian Gegenfurtner,[1] Judith Moosmüller,[1] Till Bunse,[1,2] Samuel D. Jeske,[1,2] Philipp Hagen,[1] Yi Ni,[3,4] Stephan Urban,[3,4] Ulrike Protzer1,2

[1]Institute of Virology, School of Medicine and Health, Technical University of Munich/ Helmholtz Munich, Munich, Germany

[2]German Center for Infection Research (DZIF), partner site Munich, Munich, Germany

[3]Department of Infectious Diseases, Molecular Virology, University Hospital Heidelberg, Heidelberg, Germany

[4]German Center for Infection Research (DZIF), partner site Heidelberg, Heidelberg, Germany

Fuwang Chen and Jochen M. Wettengel contributed equally to this article.

Address correspondence to Ulrike Protzer, protzer@tum.de.

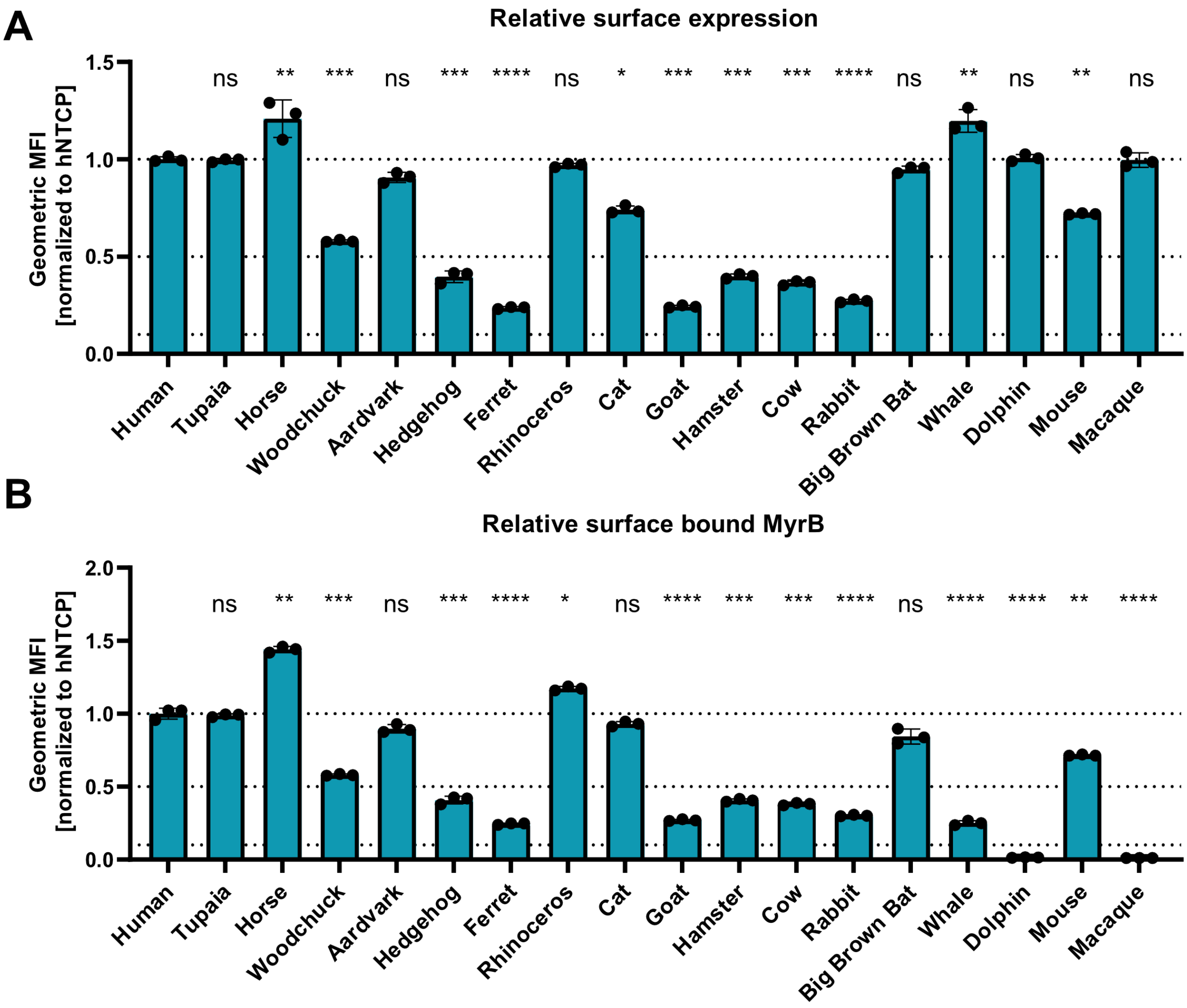

Fig. S1. NTCP surface expression and MyrB binding. HepG2 cells were transfected with IVT mRNA encoding for the indicated NTCP orthologue fused to an HA-tag. 24 h post-transfection, staining was performed using $MyrB_{atto565}$ and anti-$HA_{alexa488}$. Flow cytometry analysis of surface localized NTCP (**A**) and relative surface bound MyrB (**B**). Data was compared to hNTCP by one-way ANOVA with Dunnett's correction for multiple comparisons. Statistical significance is denoted as follows: **** p < 0,0001, *** p < 0,001, ** p < 0,01, * p < 0,05, **ns** not significant.

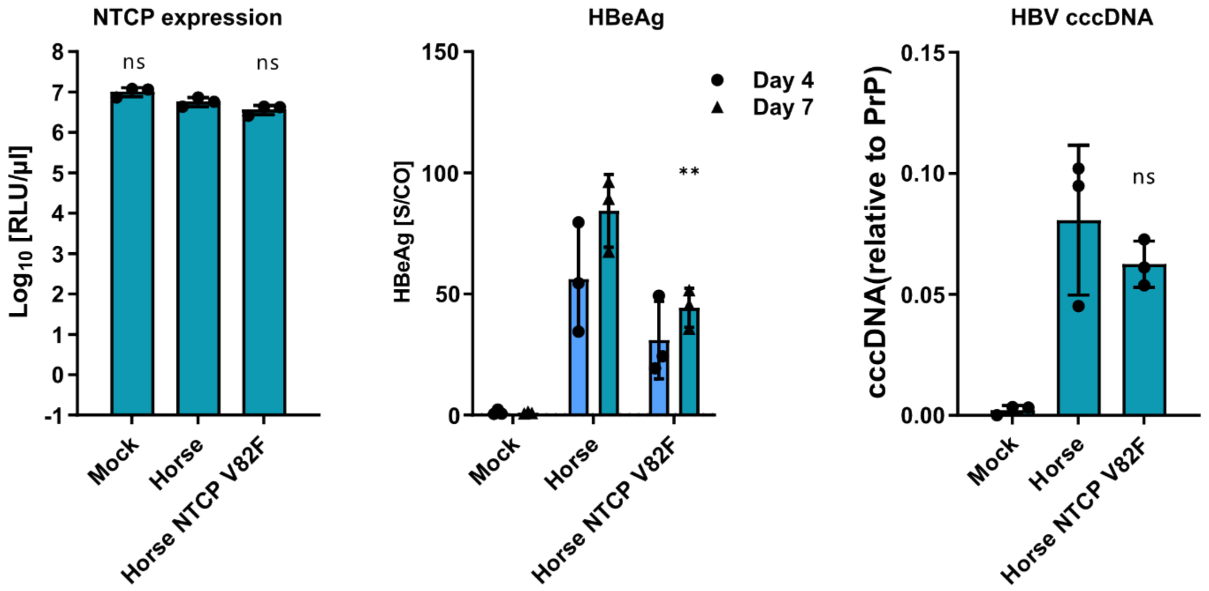

Fig. S2. Modification of aa82 of horse NTCP reduces HBV infection. HepG2 cells were transfected to express the chimeric NTCP orthologues and inoculated with HBV after 2-days pre-differentiation. The NTCP expression levels, the HBeAg, and intracellular HBV cccDNA were quantified. Data for NTCP expression and data for HBeAg and HBV cccDNA at 7 dpi were compared to horse NTCP by one-way ANOVA with Dunnett's multiple comparison correction. Statistical significance is denoted as follows: ✱✱ $p < 0{,}01$, **ns** not significant.

Table S1: Primers were used in this study (Microsynth AG, Balgach, Switzerland).

Primer Name	Sequences (5>3)
Human NTCP F	gatccgggcccagaggcccacaacgcgtctgcccc
Macaque NTCP F	gatccgggcccagaggcccacaacgcatctgc
Hamster NTCP F	gatccgggcccagaggtacacaatatctcagccccct
Woodchuck NTCP F	gatccgggcccagaggtgtacaacgtgtctgttccctc
Goat NTCP F	gatccgggcccagaggccttcaatgagtcttccccg
Cow NTCP F	gatccgggcccagaggccttcaacgaatcttccccg
Dolphin NTCP F	gatccgggcccagaggccctcaatgag
Whale NTCP F	gatccgggcccagaggccctcaatgagtctgcccag
Cat NTCP F	gatccgggcccagagccccacaatgt
Ferret NTCP F	gatccgggcccagaggctcacaacgggacggcccct
Horse NTCP F	gatccgggcccagaggcccacaatgcgtccac
Rhinozeros F	gatccgggcccagaggcccacaatgcatc
Big brown bat NTCP F	gatccgggcccagaggccctcaatg
RabbitNTCP F	gatccgggcccagaggcgcacaacgagtccg
Aardvark NTCP F	gatccgggcccagagaccctgaacacgtc
Hedgehog NTCP F	gatccgggcccagaggcccacaacgcgtctgcccccct
Tupaia NTCP F	gatccgggcccagaggcccacaacctgtccgcccca
All NTCP R	cagcgggtttaaactcaaggggcttc
Mouse NTCP F	gatccgggcccagaggcgcacaacgtatcagcc
Mouse NTCP R	cagcgggtttaaactcaaggggcttcatgctaatttgccatct
HamsterNTCP8487 F	gtcctgggcaaggtcttccgtttgaaaaatattgaggcactggccatcc
HamsterNTCP8487 R	ggatggccagtgcctcaatatttttcaaacggaagaccttgcccaggac
Goat NTCP F82V F	ctgggcaaggtcttccagctgaacaacgtc
Goat NTCP F82V R	gacgttgttcagctggaagaccttgcccag
CowNTCPF82V F	cctttggactgggcaaggtcttccagctgaataac
CowNTCPF82V R	gttattcagctggaagaccttgcccagtccaaag
